# Granulomatosis With Polyangiitis (GPA): Isolated Nasal Bridge Involvement

**DOI:** 10.7759/cureus.62911

**Published:** 2024-06-22

**Authors:** Joud Enabi, Waqar Sharif, Maneesh Mannem, Jorge L Rodriguez Vazquez, Srikanth Mukkera

**Affiliations:** 1 Internal Medicine, Texas Tech University Health Sciences Center, Odessa, USA; 2 Rheumatology, Texas Tech University Health Sciences Center, Odessa, USA

**Keywords:** vasculitis, autoimmune, isolated nasal bridge, gpa, rheumatology

## Abstract

Granulomatosis with polyangiitis (GPA) is a rare autoimmune vasculitis primarily affecting small blood vessels and presenting with systemic manifestations, including those in the kidneys and respiratory tracts. Diagnosis involves clinical assessment and specific serological tests. This paper presents the case of a 68-year-old morbidly obese woman with chronic sinusitis, hypertension, and hyperlipidemia who developed a saddle nose deformity. Despite the absence of typical respiratory and renal symptoms, her laboratory results showed positive antineutrophil cytoplasmic antibodies (ANCA) and antinuclear antibodies (ANA), with a nasal septal biopsy confirming GPA. She was treated with methotrexate and folic acid. This case underscores the variability of GPA presentations and the critical importance of early diagnosis and treatment to prevent irreversible damage.

## Introduction

Granulomatosis with polyangiitis (GPA), formerly known as Wegener's granulomatosis, is a rare but serious autoimmune disorder characterized by vasculitis, which predominantly targets small blood vessels, leading to widespread systemic manifestations. This complex disease primarily affects the kidneys as well as the upper and lower respiratory tracts, resulting in a triad of symptoms: sinusitis, pulmonary infiltrates, and glomerulonephritis. The hallmark features of GPA include necrotizing granulomatous inflammation, which can cause tissue damage and organ dysfunction if left untreated.

Despite advancements in understanding autoimmune diseases, the precise pathophysiology of GPA remains elusive. It is hypothesized that a combination of genetic predisposition and environmental triggers, such as infections, contribute to the initiation of the autoimmune response. The immune system mistakenly targets the body's own blood vessels, leading to inflammation and subsequent granuloma formation. These granulomas, composed of collections of immune cells, can obstruct blood flow and destroy normal tissue architecture, compounding the disease's complexity and severity.

Diagnosing GPA involves a multifaceted approach. Clinicians rely on a thorough clinical evaluation, which includes a detailed patient history and physical examination, to identify characteristic signs and symptoms. Additionally, specific serological tests, such as the detection of anti-neutrophil cytoplasmic antibodies (ANCAs), particularly those against proteinase 3 (PR3-ANCA), play a crucial role in confirming the diagnosis. Imaging studies and tissue biopsies may further support the clinical findings, helping to differentiate GPA from other vasculitides and inflammatory conditions.

This case report aims to highlight a unique presentation of GPA, emphasizing the diagnostic challenges and the importance of a comprehensive approach to patient management. Through this detailed exploration, we hope to contribute to the growing body of knowledge surrounding GPA and underscore the need for continued research to better understand and treat this debilitating disease.

## Case presentation

**Figure 1:**
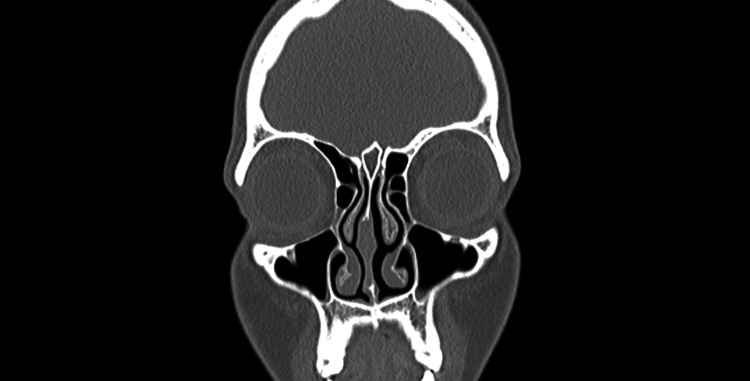
A CT scan of the sinuses did not show any abnormalities

A 68-year-old morbidly obese woman with a significant medical history of chronic sinusitis, hypertension, and hyperlipidemia noticed a new-onset deformity of her nose while getting fitted for a continuous positive airway pressure (CPAP) mask during a sleep study. Concerned about the nasal deformity, she visited the otorhinolaryngology clinic, where she was diagnosed with an acquired saddle nose deformity. Given the unusual nature of her condition, she was referred to the rheumatology department for further evaluation. She reported no symptoms such as cough, wheezing, shortness of breath, joint pain, eye symptoms, hearing loss, or hematuria, which might suggest systemic involvement. She also denied any drug use, including cocaine. Laboratory tests revealed positive ANCA at a titer of 1:80, positive myeloperoxidase (MPO) antibodies, and positive antinuclear antibodies (ANA) with a speckled pattern, titer of 1:320. A CT scan of the sinuses did not show any abnormalities, ruling out sinus involvement (Figure 1). However, a biopsy of the nasal septal cartilage revealed necrotizing chronic inflammation, focal vasculitis, and fibrotic scarring, findings that are consistent with granulomatosis with polyangiitis (GPA). Consequently, the patient was started on a treatment regimen of methotrexate and folic acid to manage her condition. Follow-up one month after the initiation of treatment showed significant improvement in her symptoms.

## Discussion

GPA is an unexplained inflammation of the medium and small arteries. Among individuals with GPA, approximately 80%-95% experience head and neck symptoms at some point [1]. Often, the initial and only signs of the disease involve ear, nose, and throat (otorhinolaryngological) symptoms. When GPA is localized solely in the head and neck region, it is termed "limited GPA," in contrast to the more widespread form known as "generalized GPA." Generalized GPA involves systemic vasculitis affecting organs like the kidneys and lungs, along with symptoms like fever and weakness [2]. The limited GPA phenotype is more common in young female patients, tends to recur, and can be challenging to manage with medical therapy. Nasal involvement is a recognized feature of limited GPA, typically beginning in the septum area supplied by the Kiesselbach plexus and spreading to the paranasal sinuses [3]. In our specific case, the patient's symptoms are centered around the sinonasal region and do not involve systemic issues. Nasal endoscopy revealed a sizable perforation in the nasal septum and a transparent mass in the right nasal fossa. The presence of a transparent mass in the middle meatus is usually associated with an inflammatory condition like nasal polyps, although the simultaneous presence of a nasal septum perforation (NSP) is uncommon. When diagnosing NSP, it is crucial to rule out previous cocaine use, which can lead to nasal septum destruction [4]. NSP is a common feature of autoimmune disorders, and in cases like ours, granulomatous damage to nasal cartilage can result in septum perforation and potentially lead to a saddle-nose deformity [5]. A recent review by Guntpalli et al. found that GPA and relapsing polychondritis are commonly associated with nasal septum perforation, accounting for nearly 80% of reported cases in the literature [5]. A prompt and accurate diagnosis of GPA is essential to initiating timely treatment and preventing irreversible organ damage. Diagnosing GPA can be challenging because it presents various clinical manifestations and can be difficult to differentiate from malignancies, infectious diseases (e.g., tuberculosis, aspergillosis, leishmaniasis), and inflammatory conditions (e.g., sarcoidosis) [6]. Diagnosis often relies on the presence of distinct ANCA markers and biopsies of affected organs. In GPA, cytoplasmic ANCA (c-ANCA) is highly specific, while peri-nuclear ANCA (p-ANCA) may be associated with other autoimmune conditions [7]. The sensitivity and specificity of c-ANCA testing are typically high during the active phases of the disease. However, in localized GPA of the ear, nose, and throat, c-ANCA levels may be less elevated. Biopsy becomes more crucial when ANCA testing is inconclusive, particularly in limited GPA where c-ANCA's predictive value is lower. Histological findings in GPA include vasculitis, granulomas, giant cells, and necrosis. The accuracy of a tissue biopsy varies depending on the location of the active disease [8]. In cases involving the nasal region, nasal biopsies are recommended, although they can have a relatively high rate of false negatives compared to biopsies in other affected areas. While nasal symptoms are highly specific for GPA in the head and neck, GPA can also affect other head and neck sites. Audiovestibular symptoms, often underestimated, may occur in a significant percentage of GPA patients [9]. These symptoms typically involve sensorineural hearing loss, although conductive hearing loss can occur in cases involving the middle ear or Eustachian tube dysfunction. Audiometric patterns of GPA-related hearing loss are typically flat and can coexist with age-related high-frequency loss. Sudden sensorineural hearing loss can also be an initial symptom of GPA and is often an indicator of disease worsening, necessitating specific treatment [10]. Awareness of GPA is crucial for clinical otolaryngologists due to its varied clinical manifestations. A prompt diagnosis is essential to initiating appropriate therapy and preventing the progression of the disease to renal or lung failure.

## Conclusions

This case of a 68-year-old female presenting with acquired saddle nose deformity highlights the critical importance of considering GPA in patients with sinonasal abnormalities, particularly in those with a history of chronic sinusitis. The patient's positive serological markers (ANCA) and biopsy findings were pivotal in confirming the diagnosis of GPA, despite the absence of more common systemic symptoms such as renal or pulmonary involvement. This case underscores the necessity of a thorough clinical and diagnostic evaluation in patients with atypical presentations to enable early diagnosis and treatment of GPA, thereby preventing potentially severe complications. The initiation of methotrexate and folic acid treatment aims to manage the autoimmune response and mitigate further disease progression. Awareness and early recognition of GPA's diverse manifestations can significantly improve patient outcomes, particularly in cases with localized disease presentations.
